# miR‐155‐5p inhibition rejuvenates aged mesenchymal stem cells and enhances cardioprotection following infarction

**DOI:** 10.1111/acel.13128

**Published:** 2020-03-20

**Authors:** Yimei Hong, Haiwei He, Guojun Jiang, Hao Zhang, Wuyuan Tao, Yue Ding, Dongsheng Yuan, Jing Liu, Huimin Fan, Fang Lin, Xiaoting Liang, Xin Li, Yuelin Zhang

**Affiliations:** ^1^ Guangdong Provincial People's Hospital Guangdong Academy of Medical Sciences School of Medicine South China University of Technology Guangzhou China; ^2^ Department of Emergency Medicine Guangdong Provincial People's Hospital Guangdong Academy of Medical Sciences Guangzhou China; ^3^ Department of Emergency and Critical Care Medicine Guangdong Provincial People's Hospital Guangdong Academy of Medical Sciences Guangzhou China; ^4^ Faculty of Pharmacy Bengbu Medical College Bengbu China; ^5^ Department of Organ Transplantation Changzheng Hospital Second Military Medical University Shanghai China; ^6^ Clinical Translational Medical Research Center Shanghai East Hospital Tongji University School of Medicine Shanghai China; ^7^ Institute of Regenerative Medicine Shanghai East Hospital Tongji University School of Medicine Shanghai China

**Keywords:** mesenchymal stem cells, miR‐155‐5p, myocardial infarction, rejuvenation, senescence

## Abstract

Aging impairs the functions of human mesenchymal stem cells (MSCs), thereby severely reducing their beneficial effects on myocardial infarction (MI). MicroRNAs (miRNAs) play crucial roles in regulating the senescence of MSCs; however, the underlying mechanisms remain unclear. Here, we investigated the significance of miR‐155‐5p in regulating MSC senescence and whether inhibition of miR‐155‐5p could rejuvenate aged MSCs (AMSCs) to enhance their therapeutic efficacy for MI. Young MSCs (YMSCs) and AMSCs were isolated from young and aged donors, respectively. The cellular senescence of MSCs was evaluated by senescence‐associated β‐galactosidase (SA‐β‐gal) staining. Compared with YMSCs, AMSCs exhibited increased cellular senescence as evidenced by increased SA‐β‐gal activity and decreased proliferative capacity and paracrine effects. The expression of miR‐155‐5p was much higher in both serum and MSCs from aged donors than young donors. Upregulation of miR‐155‐5p in YMSCs led to increased cellular senescence, whereas downregulation of miR‐155‐5p decreased AMSC senescence. Mechanistically, miR‐155‐5p inhibited mitochondrial fission and increased mitochondrial fusion in MSCs via the AMPK signaling pathway, thereby resulting in cellular senescence by repressing the expression of Cab39. These effects were partially reversed by treatment with AMPK activator or mitofusin2‐specific siRNA (Mfn2‐siRNA). By enhancing angiogenesis and promoting cell survival, transplantation of anti‐miR‐155‐5p‐AMSCs led to improved cardiac function in an aged mouse model of MI compared with transplantation of AMSCs. In summary, our study shows that miR‐155‐5p mediates MSC senescence by regulating the Cab39/AMPK signaling pathway and miR‐155‐5p is a novel target to rejuvenate AMSCs and enhance their cardioprotective effects.

## Graphical abstract

Inhibition of miR‐155‐5p, via the Cab39/AMPK signaling pathway, rejuvenates aged MSCs by regulating mitochondrial dynamics. Inhibition of miR‐155‐5p rejuvenated AMSCs and increased cell survival and angiogenesis in infarcted mouse hearts, thereby promoting the cardioprotective effects of AMSCs.

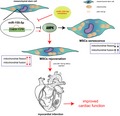

## 1 INTRODUCTION

Despite the existing treatments, including percutaneous coronary intervention and coronary artery bypass grafting, myocardial infarction (MI) is still the main cause of morbidity and mortality worldwide, particularly in elderly patients (Zhang et al., 2018). Over the last few decades, mesenchymal stem cell (MSC)‐based therapy has emerged as a novel alternative treatment for MI (Kim et al., 2018; Teerlink et al., 2017). Accumulating evidence has demonstrated that transplantation of MSCs can attenuate cardiac remodeling and improve heart function recovery following MI by inhibiting cardiomyocyte apoptosis, increasing angiogenesis, and rejuvenating cardiac muscle cells (Liao et al., 2019; Zhang et al., 2017, 2019). However, the functions of MSCs dramatically decline with aging, as evidenced by increased cellular senescence, impaired proliferative capacity, and decreased paracrine effects, thereby heavily limiting their cardioprotective effects following MI (Liu et al., 2014; Zhang et al., 2018). Therefore, exploring a novel strategy to rejuvenate aged MSCs (AMSCs) to enhance their therapeutic effects for elderly patients with MI is urgently needed.

MicroRNAs (miRNAs), a class of ~21–23 nucleotide long noncoding RNAs, are critical repressors of gene expression by virtue of binding to the 3′‐untranslated region (UTR) of target mRNAs (Xu et al., 2018). miRNAs have been reported to be involved in mediating multiple biological processes of stem cells, including cell division, differentiation, and survival (Wang et al., 2018; Zhou et al., 2019). Recently, increasing evidence has demonstrated that miRNAs play crucial roles in regulating the cellular senescence of MSCs (Meng et al., 2018; Yoo, Kim, Jung, Lee, & Kim, 2014). miR‐495 targets Bmi‐1 and induces MSC senescence, as evidenced by enhanced β‐galactosidase activity and reduced cell proliferation (Li et al., 2017). Moreover, miR‐29c‐3p is upregulated during the replicative senescence of MSCs and suppresses their functions by targeting CNOT6 to activate the p53‐p21 and p16‐pRB pathways (Shang et al., 2016). It has been reported that miR‐155‐5p is significantly enhanced with age in bone marrow‐derived extracellular vesicles (Davis et al., 2017), which prompted us to study the relationship of miR‐155‐5p and aging. Furthermore, miR‐155‐5p was found to be elevated in dermal MSCs of psoriatic patients, indicating that miR‐155‐5p could impair the functions of MSCs (Hou et al., 2016). However, whether miR‐155‐5p regulates the cellular senescence of MSCs has not been determined.

Mitochondria undergo mitochondrial fission and fusion to regulate cell physiology. Mitochondrial fission, mediated by dynamin‐related protein 1 (Drp1) and fission 1 (Fis1), results in small round mitochondria; mitochondrial fusion, controlled by Mitofusin 1 (Mfn1), Mitofusin 2 (Mfn2), and optic atrophy protein 1 (OPA1), generates long mitochondrial tubules (van der Bliek, Shen, & Kawajiri, 2013). It is well known that the imbalance between mitochondrial fusion and fission is closely associated with cellular senescence (Nishimura et al., 2018). TGF‐β induces vascular progenitor cell senescence by stimulating mitochondrial fusion (He et al., 2019). However, whether miR‐155‐5p mediates MSC senescence by regulating mitochondrial dynamics and the potential underlying mechanisms remain to be addressed.

In the current study, we aimed to investigate the role of miR‐155‐5p in regulating the senescence of MSCs and explored the related molecular mechanisms. Furthermore, we also examined whether inhibition of miR‐155‐5p could rejuvenate AMSCs and improve cardioprotection when AMSCs were transplanted into a mouse model of MI.

## 2 RESULTS

### 2.1 AMSCs exhibit increased cellular senescence

**Figure 1:**
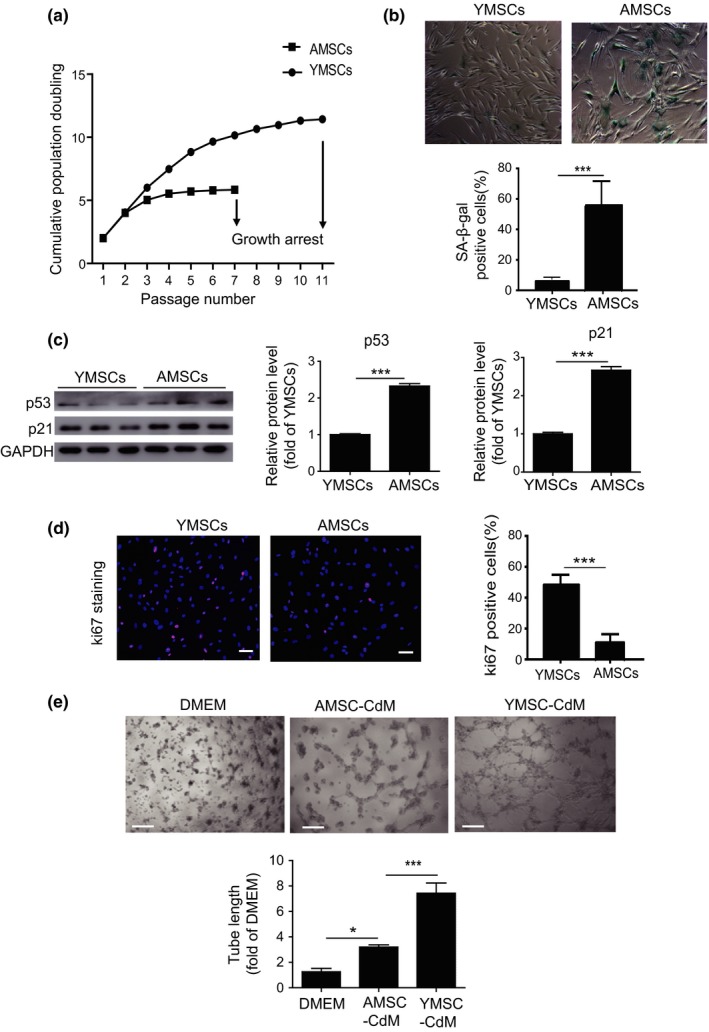
AMSCs exhibited increased cellular senescence. (a) Cell growth curves demonstrated the decreased proliferative ability of AMSCs compared to YMSCs. (b) Representative images of SA‐β‐gal staining and quantitative analysis of SA‐β‐gal‐positive cells in YMSCs and AMSCs. Scale bar = 200 μm. (c) Western blotting and quantitative analysis of the expression levels of p53 and p21 in YMSCs and AMSCs. (d) Immunostaining of the proliferation marker Ki67 and quantitative analysis of Ki67‐positive cells in YMSCs and AMSCs. Scale bar = 100 μm. (e) Representative images of tube formation and analysis of tube length in HUVECs treated with DMEM, YMSC‐CdM, or AMSC‐CdM. Scale bar = 200 μm. Data are expressed as the mean ± *SEM*. *n* = 3. *p < .05; ***p < .001

We first examined the surface antigens of young MSCs (YMSCs) and AMSCs. Flow cytometry analysis showed that both YMSCs and AMSCs had similar surface markers and were positive for CD73, CD90, and CD105 and negative for CD31 and CD45 (Figure S1A). Next, we evaluated the differentiation capacity of YMSCs and AMSCs. The results showed that both YMSCs and AMSCs differentiated into adipocytes and osteocytes, as confirmed by oil red O staining and alizarin red staining, respectively (Figure S1B,C). In addition, AMSCs exhibited increased adipogenic capacity and decreased osteogenic capacity compared with YMSCs, suggesting that the functions of AMSCs were impaired (Figure S1B,C). A previous study showed that senescent MSCs display increased adipogenic and decreased osteogenic differentiation capacities (Ma et al., 2018); thus, we examined the cellular senescence of YMSCs and AMSCs. Cell growth curves showed that AMSCs exhibited lower proliferative ability and arrested at passage 7, whereas YMSCs continued growing until passage 11 (Figure 1a). Furthermore, compared with YMSCs, AMSCs exhibited increased levels of senescence‐associated β‐galactosidase (SA‐β‐gal) activity (Figure 1b) and increased expression levels of p53 and p21 protein (Figure 1c). Additionally, Ki67 immunostaining showed a lower proliferative ability of AMSCs than YMSCs (Figure 1d). A wound healing assay demonstrated decreased migration ability of AMSCs compared with YMSCs (Figure S1D). Promoting neovascularization is one of the major mechanisms underlying MSC‐based therapy for MI. We therefore evaluated the angiogenic capacity of conditioned medium (CdM) from YMSCs and AMSCs. As shown in Figure 1e, tube length was significantly increased in the CdM from MSCs compared with DMEM. Notably, compared with YMSCs‐CdM treatment, AMSCs‐CdM treatment presented decreased endothelial network formation capacity (Figure 1e). Taken together, these data show that AMSCs exhibit increased cellular senescence.

### 2.2 miR‐155‐5p mediates cellular senescence of MSCs

**Figure 2:**
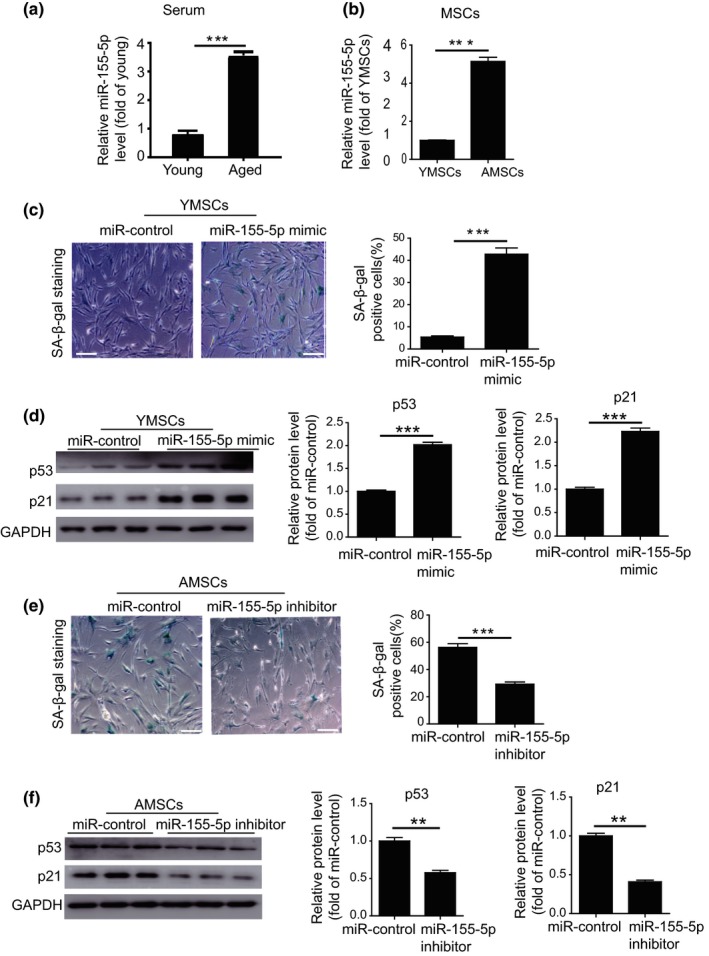
miR‐155‐5p mediated the cellular senescence of MSCs. (a) The expression level of miR‐155‐5p was measured in the serum from aged and young donors. (b) The expression level of miR‐155‐5p was measured in AMSCs and YMSCs. (c) Representative images of SA‐β‐gal staining and quantitative analysis of SA‐β‐gal‐positive YMSCs transfected with miR‐control or miR‐155‐5p mimic. Scale bar = 200 μm. (d) Western blotting and quantitative analysis of the expression levels of p53 and p21 in YMSCs transfected with miR‐control or miR‐155‐5p mimic. (e) Representative images of SA‐β‐gal staining and quantitative analysis of SA‐β‐gal‐positive AMSCs transfected with miR‐control or miR‐155‐5p inhibitor. Scale bar = 200 μm. (f) Western blotting and quantitative analysis of the expression levels of p53 and p21 in AMSCs transfected with miR‐control or miR‐155‐5p inhibitor. Data are expressed as the mean ± *SEM*. *n* = 3. **p < .01; ***p < .001

To investigate whether miR‐155‐5p regulates MSC senescence, we carried out qRT‐PCR to measure miR‐155‐5p expression in serum from young donors (*n* = 7, 31.7 ± 3.6 years old) and aged donors (*n* = 7, 65.3 ± 2.4 years old). Compared with young donors, miR‐155‐5p levels were dramatically increased in aged donors (Figure 2a). Additionally, miR‐155‐5p levels were much higher in AMSCs than in YMSCs (Figure 2b), suggesting that miR‐155‐5p expression is positively correlated with the cellular senescence of MSCs. Next, we examined the role of miR‐155‐5p in MSC senescence. Treating YMSCs with the miR‐155‐5p mimic caused a significant increase in miR‐155‐5p (Figure S2A). Compared with miR‐control treatment, miR‐155‐5p mimic treatment greatly enhanced the level of SA‐β‐gal activity (Figure 2c) and the expression levels of p21 and p53 proteins (Figure 2d) in YMSCs. In addition, miR‐155‐5p mimic treatment significantly reduced the proliferation of YMSCs, as evidenced by the reduced Ki67‐positive cells (Figure S2B). Moreover, miR‐155‐5p mimic treatment also downregulated the angiogenic capacity of CdM from YMSCs, as shown by the reduction in endothelial network formation (Figure S2C). To further verify the role of miR‐155‐5p in the regulation of MSC senescence, we treated AMSCs with a miR‐155‐5p inhibitor. Administration of the miR‐155‐5p inhibitor led to a significant reduction in the miR‐155‐5p level (Figure S2D), the level of SA‐β‐gal activity (Figure 2e) and the expression levels of p21 and p53 protein (Figure 2f) in AMSCs. Furthermore, miR‐155‐5p inhibitor treatment significantly improved the proliferative capacity (Figure S2E) and the angiogenic capacity of CdM from AMSCs (Figure S2F). Collectively, these findings suggest that miR‐155‐5p mediates the cellular senescence of MSCs.

### 2.3 miR‐155‐5p induces cellular senescence of MSCs by regulating mitochondrial dynamics

**Figure 3:**
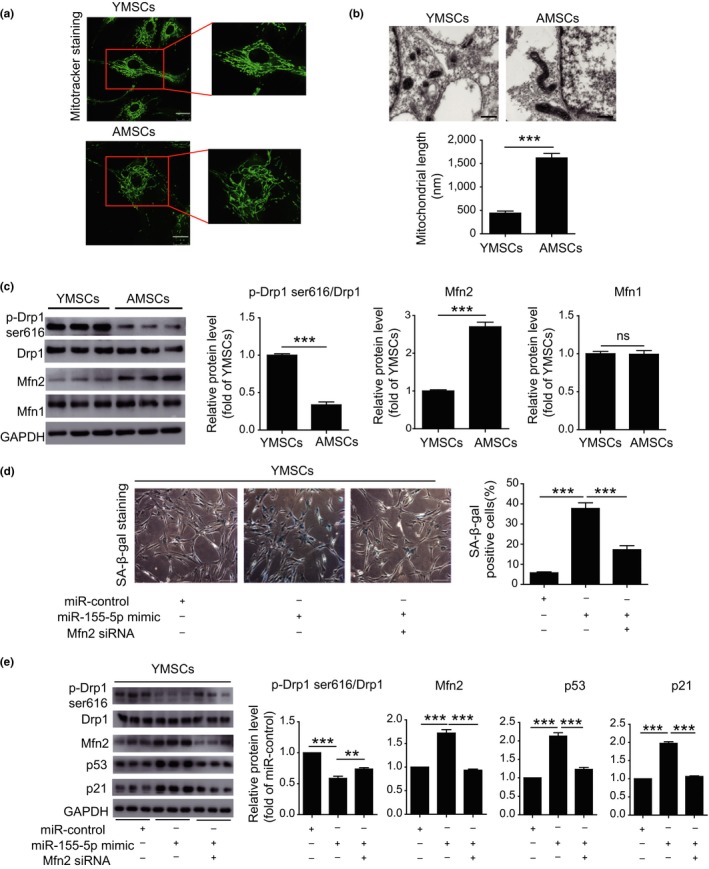
miR‐155‐5p accelerated the cellular senescence of MSCs by regulating mitochondrial dynamics. (a) Representative images of MitoTracker staining of YMSCs and AMSCs. Scale bar = 25μm. (b) Representative images of mitochondria in ECM and quantitative analysis of mitochondrial length in AMSCs and YMSCs. Scale bar = 500 nm. (c) Western blotting and quantitative analysis of the expression levels of p‐Drp1 (Ser616), Mfn1 and Mfn2 in AMSCs and YMSCs. (d) Representative images of SA‐β‐gal staining and quantitative analysis of SA‐β‐gal‐positive YMSCs transfected with miR‐control, miR‐155‐5p mimic, or miR‐155‐5p mimic + Mfn2‐siRNA. Scale bar = 200 μm. (e) Western blotting and quantitative analysis of the expression levels of p‐Drp1 (Ser616), Mfn1 and Mfn2 in YMSCs transfected with miR‐control, miR‐155‐5p mimic, or miR‐155‐5p mimic + Mfn2‐siRNA. Data are expressed as the mean ± *SEM*. *n* = 3. **p < .01; ***p < .001. ns = not significant

Our previous study showed that mitochondrial fusion contributes to replicative senescence of MSCs (Li et al., 2019); thus, we explored whether miR‐155‐5p induces cellular senescence of MSCs by regulating mitochondrial dynamics. We first examined the mitochondrial morphology in YMSCs and AMSCs. As shown in Figure 3a, MitoTracker staining showed that YMSCs had small tubular mitochondria, whereas AMSCs had large tubular mitochondria (Figure 3a). Furthermore, we examined the mitochondrial morphology in YMSCs and AMSCs using transmission electron microscope (TEM). As shown in Figure 3b, the mitochondria in AMSCs were much longer than YMSCs (Figure 3b). Western blotting analysis showed that compared with those in YMSCs, the expression level of p‐Drp1 (Ser616) was greatly decreased, whereas the expression level of Mfn2 was increased in AMSCs (Figure 3c). In addition, there was no difference in Mfn1 expression between YMSCs and AMSCs (Figure 3c). These results suggest that an imbalance of mitochondrial dynamics is associated with MSC senescence. Next, we treated YMSCs with a miR‐155‐5p mimic and found that miR‐155‐5p mimic treatment enhanced the level of SA‐β‐gal activity along with an increase in the expression of Mfn2, p53, and p21 and a reduction in p‐Drp1 (Ser616) in YMSCs (Figure 3d,e). Remarkably, these effects were largely reversed by Mfn2‐siRNA treatment (Figure 3d,e), indicating that miR‐155‐5p induced MSC senescence by activating mitochondrial fusion. Furthermore, miR‐155‐5p inhibitor treatment alleviated the senescence of AMSCs (Figure S3A), increased the expression of p‐Drp1 and reduced the expression of Mfn2, p53, and p21 (Figure S3B). Notably, the alleviation of senescence in AMSCs by the miR‐155‐5p inhibitor was partially reversed by the Drp1 inhibitor Mdivi 1, as evidenced by increased SA‐β‐gal activity and the increased expression of p53 and p21 (Figure S3A,B). Collectively, these observations suggest that miR‐155‐5p induces cellular senescence of MSCs by regulating mitochondrial dynamics.

### 2.4 miR‐155‐5p regulates mitochondrial dynamics via the Cab39/AMPK signaling pathway

**Figure 4:**
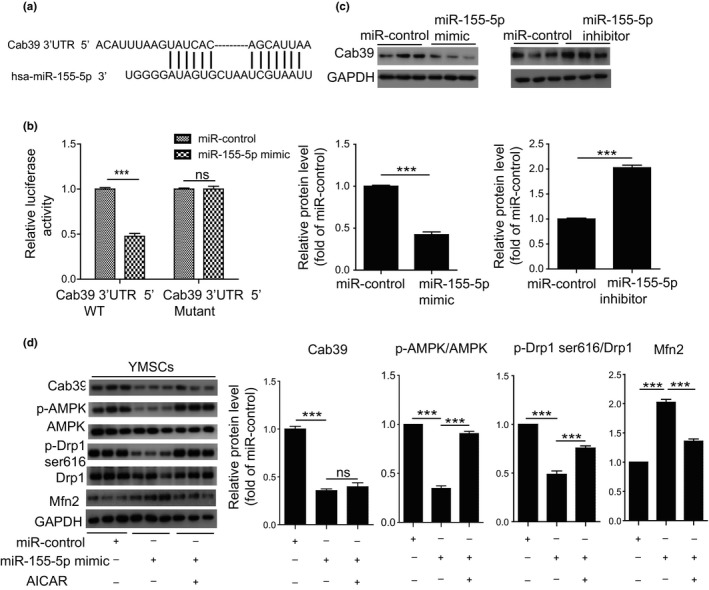
miR‐155‐5p regulated mitochondrial dynamics via the Cab39/AMPK signaling pathway. (a) The potential binding sites for miR‐155‐5p on the 3′UTR of Cab39. (b) 293T cells were cotransfected with miR‐155‐5p mimic or miRNA control and with a luciferase reporter vector containing WT or mutant 3′UTR of Cab39. (c) Western blotting and quantitative analysis of the expression level of Cab39 in YMSCs transfected with a scrambled miRNA control, miR‐155‐5p mimic or miR‐155‐5p inhibitor. (d) Western blotting and quantitative analysis of the expression levels of Cab39, p‐AMPK, AMPK, p‐Drp1 (Ser616), Drp1, and Mfn2 in YMSCs transfected with miR‐control, miR‐155‐5p mimic or miR‐155‐5p mimic + AICAR. Data are expressed as the mean ± *SEM*. *n* = 3. ***p < .001. ns = not significant

Our previous studies showed that the AMPK signaling pathway plays a critical role in regulating mitochondrial dynamics; thus, we aimed to determine whether miR‐155‐5p regulates mitochondrial dynamics via the AMPK signaling pathway (He et al., 2019; Li et al., 2019). We used TargetScan (http://www.targetscan.org/) to predict the target genes of miR‐155‐5p and found a potential binding sequence in the 3′UTR of calcium‐binding protein 39 (Cab39) (Figure 4a). Cab39 is a component of the trimeric liver kinase B1 (LKB1)‐STRAD‐Cab39 complex and regulates the activity of LKB1 and thus activates the phosphorylation of AMPK (Xu et al., 2019). A dual‐luciferase reporter gene assay demonstrated that the miR‐155‐5p mimic significantly reduced the luciferase activity of the Cab39 wild‐type (WT) reporter but had no impact on the luciferase activity of the Cab39 mutant reporter (Figure 4b). To evaluate whether endogenous miR‐155‐5p regulated Cab39, we transfected YMSCs and AMSCs with wild‐type pGL3‐Cab39‐3′‐UTR luciferase reporter, respectively, and then examined the luciferase activity. As shown in Figure S4, compared with YMSCs, the luciferase activity was dramatically reduced in AMSCs, indicating a negative relationship between endogenous miR‐155‐5p and Cab39 (Figure S4). MiR‐155‐5p mimic treatment significantly reduced the mRNA level of Cab39 in MSCs (Figure S5A). Western blotting experiments showed that the miR‐155‐5p mimic greatly reduced the expression of Cab39, whereas the miR‐155‐5p inhibitor enhanced the expression of Cab39 in YMSCs, suggesting that the expression level of Cab39 negatively correlated with the level of miR‐155‐5p in MSCs (Figure 4c). Next, we examined the expression levels of Cab39 and p‐AMPK in YMSCs and AMSCs. Compared with those in YMSCs, the expression levels of Cab39 and p‐AMPK were robustly decreased in AMSCs (Figure S5B). Furthermore, treatment with the miR‐155‐5p mimic dramatically downregulated the expression of Cab39, p‐AMPK, and p‐Drp1 (Ser616) and upregulated the expression of Mfn2 (Figure 4d). Furthermore, treatment with AICAR, an AMPK activator, partially reversed the downregulation of p‐AMPK and p‐Drp1 and the upregulation of Mfn2 induced by the miR‐155‐5p mimic (Figure 4d). To further verify the role of Cab39 in miR‐155‐5p induced MSC senescence, we overexpressed Cab39 which lacks the miR‐155‐5p target site in YMSCs and then treated the cells with miR‐155‐5p mimic. As shown in Figure S5, overexpressed Cab39 rescued miR‐155‐5p‐induced YMSC senescence (Figure S6A). Moreover, overexpressed Cab39 inhibited miR‐155‐5p‐induced mitochondrial fusion in YMSCs (Figure S6B). Overall, miR‐155‐5p regulates mitochondrial dynamics in MSCs by targeting the Cab39/AMPK signaling pathway.

### 2.5 Transplantation of anti‐miR‐155‐5p‐AMSCs improves cardiac function following infarction in aged mice

**Figure 5:**
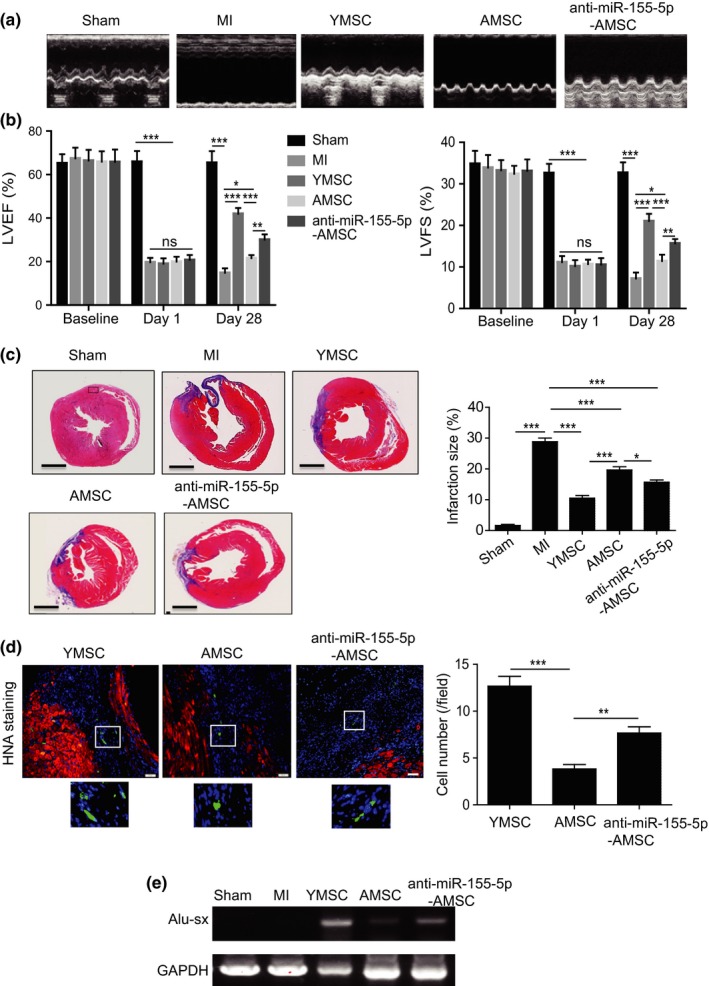
Transplantation of anti‐miR‐155‐5p‐AMSCs improved heart function following infarction in aged mice. (a) Representative echocardiography images taken 28 days after MI in aged mice that received injections of PBS, YMSCs, AMSCs, or anti‐miR‐155‐5p‐AMSCs or control mice. (b) The LVEF and LVFS were evaluated at baseline (before MI), 1 and 28 days in control or aged mice with MI that received injections of PBS, YMSCs, AMSCs or anti‐miR‐155‐5p‐AMSCs. (c) Representative images of Masson's trichrome staining and quantitative analysis of infarction size in control or aged mice with MI that received injections of PBS, YMSCs, AMSCs, or anti‐miR‐155‐5p‐AMSCs. Scale bar = 2.5 mm. (d) Representative images of HNA staining and quantitative analysis of cell survival in aged mice that received injections of YMSCs, AMSCs or anti‐miR‐155‐5p‐AMSCs at 28 days post‐MI. Scale bar = 50 μm. (e) Representative PCR image of Alu‐sx in the ischemic heart tissue in aged mice that received injections of YMSCs, AMSCs or anti‐miR‐155‐5p‐AMSCs at 28 days post‐MI. Data are expressed as the mean ± *SEM*. *n* = 6–7. **p < .05; **p < .01; ***p < .001

To examine whether inhibition of miR‐155‐5p in AMSCs can improve the therapeutic effects of MSCs, we transplanted anti‐miR‐155‐5p‐AMSCs into infarcted mouse hearts. The heart function of mice from different groups was measured by echocardiography at baseline (before MI), 1, and 28 days post‐MI. Representative images of echocardiography were taken at 28 days after MI in mice (Figure 5a). Echocardiography revealed that compared with those in the control group, left ventricle ejection fraction (LVEF) and fraction shorting (LVFS) were robustly reduced on 1 day post‐MI in the MI group, YMSC group, AMSC group, and anti‐miR‐155‐5p‐AMSC group, indicating that the mouse model of MI was successfully established (Figure 5b). Notably, no difference in LVEF and LVFS was observed among the MI group, YMSCs group, AMSCs group, and anti‐miR‐155‐5p‐AMSCs group, suggesting that a similar degree of MI was induced in all surgery groups (Figure 5b). However, at 28 days post‐MI, the LVEF and LVFS were greatly enhanced in all MSC‐transplanted groups compared with the MI group (Figure 5b). Furthermore, the LVEF and LVFS were significantly reduced in the AMSC group compared with the YMSC group but were partially restored in the anti‐miR‐155‐5p‐AMSC group, indicating that anti‐miR‐155‐5p‐AMSCs were superior to AMSCs at improving heart function following MI (Figure 5b).

Similarly, the infarct size determined by Masson's trichrome staining was much higher in the AMSC group than in the YMSC group at 28 days post‐MI. However, the infarct size was significantly reduced in the anti‐miR‐155‐5p‐AMSCs group compared with the AMSC group (Figure 5c). Next, we examined the survival of MSCs at 28 days post‐transplantation. Human nuclear antigen (HNA) staining showed that although the number of surviving MSCs was the highest in heart tissue from the YMSC group, MSC survival was much higher in the anti‐miR‐155‐5p‐AMSC group than in the AMSC group (Figure 5d). To further confirm the survival of transplanted MSCs in ischemic heart tissue of mice, we detected the human repeat sequences Alu‐sx in heart tissue using PCR. As shown in Figure 5f, Alu‐sx was detected in all MSC groups, but not in the sham group and MI group (Figure 5f). Notably, the expression of Alu‐sx was greatly enhanced in the anti‐miR‐155‐5p‐AMSC group than in the AMSC group (Figure 5f). Taken together, these data show that inhibition of miR‐155‐5p in AMSCs can improve cardioprotection following MI in mice.

### 2.6 Anti‐miR‐155‐5p‐AMSC transplantation inhibits cardiomyocyte apoptosis and enhances angiogenesis in infarcted mouse hearts

**Figure 6:**
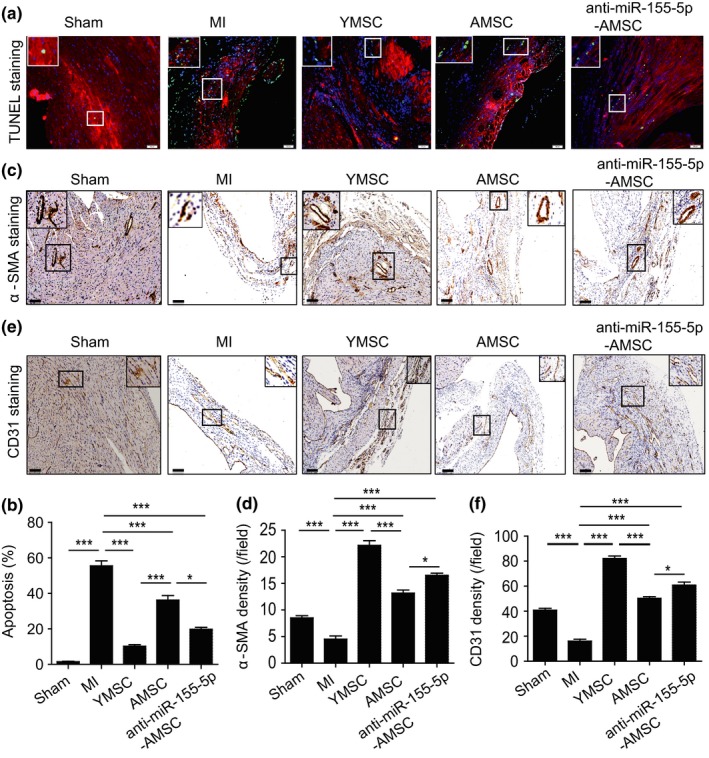
Transplantation of anti‐miR‐155‐5p‐AMSCs inhibited apoptosis of cardiomyocytes and enhanced angiogenesis following infarction in aged mice. (a) Representative images of TUNEL staining of heart tissue from control or aged mice with MI that received injections of PBS, YMSCs, AMSCs, or anti‐miR‐155‐5p‐AMSCs. Scale bar = 100 μm. (b) Quantitative analysis of the apoptosis of cardiomyocytes among the different groups. (c) Representative images of α‐SMA staining of heart tissue from control or aged mice with MI that received injections of PBS, YMSCs, AMSCs, or anti‐miR‐155‐5p‐AMSCs. Scale bar = 200 μm. (d) Quantitative analysis of the density of arterioles among the different groups. (e) Representative images of CD31 staining of heart tissue from control or aged mice with MI that received injections of PBS, YMSCs, AMSCs, or anti‐miR‐155‐5p‐AMSCs. Scale bar = 200 μm. (f) Quantitative analysis of the density of capillaries among the different groups. Data are expressed as the mean ± *SEM*. *n* = 6–7. **p < .05; **p < .01; ***p < .001

To evaluate the anti‐apoptotic effects of MSC transplantation, the apoptosis of cardiomyocytes in the ischemic area was determined by terminal deoxynucleotidyl transferase dUTP nick end labeling (TUNEL) staining. Compared with that in the MI group, the apoptosis of the cardiomyocytes was greatly reduced in all MSC‐transplanted groups, and the apoptosis was the lowest in the YMSC group (Figure 6a,b). Moreover, fewer apoptotic cardiomyocytes were observed in the hearts from the anti‐miR‐155‐5p‐AMSC group than in the AMSC group (Figure 6a,b). To determine the angiogenic effects of MSC transplantation, the arteriole densities and capillary densities were examined by α‐SMA staining and CD31 staining in mouse hearts at 28 days post‐transplantation, respectively. As shown in Figure 6c,d, the arteriole density was significantly increased in all MSC‐treated groups compared with the MI group, and the arteriole density was the highest in the YMSC group (Figure 6c,d). Notably, more arterioles were found in the anti‐miR‐155‐5p‐AMSC group than in the AMSC group (Figure 6c,d). Consistent with these findings, a similar result was shown in the capillary densities from the different MSC‐treated groups. The capillary density was the highest in the heart tissue from the YMSC group, and more capillaries were formed in the anti‐miR‐155‐5p‐AMSC group than in the AMSC group (Figure 6e,f). Collectively, these findings suggest that anti‐miR‐155‐5p‐AMSC transplantation inhibits cardiomyocyte apoptosis and enhances angiogenesis in infarcted mouse hearts.

## 3 DISCUSSION

The current study presented several major findings. First, miR‐155‐5p was upregulated in AMSCs and regulated the cellular senescence of MSCs. Second, miR‐155‐5p mediated MSC senescence by regulating mitochondrial dynamics. Third, miR‐155‐5p regulated mitochondrial dynamics by targeting the Cab39/AMPK signaling pathway. Finally, inhibition of miR‐155‐5p rejuvenated AMSCs and increased cell survival and angiogenesis in infarcted mouse hearts, thereby promoting the cardioprotective effects of AMSCs.

Over the past few decades, transplantation of MSCs has demonstrated promising results on MI recovery in animal studies and early clinical trials due to the availability of numerous sources, multi‐lineage potential, and immunoprivileged status of these cells (Florea et al., 2017; Lu et al., 2019; Xiao et al., 2018). Despite the beneficial effects of allogeneic transplantation of MSCs for MI in the early days post‐transplantation, the long‐term effects of allogeneic MSCs to preserve heart function are heavily limited compared with syngeneic MSCs due to low cell survival caused by immunorejection (Huang et al., 2010). Therefore, using autologous MSCs can obviate this concern and enhance the protective effects of the cells. However, autologous MSCs isolated from older patients have become senescent, leading to decreased cell homeostatic and regenerative capacity (Song et al., 2017). Furthermore, compared with the transplantation of YMSCs, transplantation of AMSCs exhibits a lower cardiac repair capacity following MI in rats (Zhai et al., 2016). Consistent with these observations, in the current study, MSCs collected from aging donors displayed an increased level of SA‐β‐gal activity and decreased proliferative and differentiation capacity. Furthermore, the paracrine effects of the AMSCs were also dramatically decreased. These results suggested that the cells were senescent. We also observed that transplantation of AMSCs had a lower therapeutic efficacy for MI in mice compared with transplantation of YMSCs. Indeed, rejuvenating aged MSCs to increase their therapeutic efficacy for cardiovascular diseases has attracted a lot of attention, and several novel strategies have been explored to rejuvenate aged MSCs, including gene modification (Liang et al., 2019; Song et al., 2017) and pharmacological pretreatment (Fang et al., 2018). However, the potential mechanisms underlying MSC senescence remain unclear.

Recently, a variety of miRNAs have been reported to be involved in regulating MSC senescence via multiple pathways (Lei et al., 2017). Overexpression of miR‐195 could induce cellular senescence of MSCs by repressing telomerase reverse transcriptase (Tert), leading to a reduction in their regenerative ability. Furthermore, deletion of miR‐195 can reverse MSC aging and thereby improve cardiac repair following infarction in mice (Okada et al., 2016). Downregulation of miR‐543 and miR‐590‐3p induces the senescence phenotype of MSCs by inducing AIMP3/p18 overexpression, which compromises the adipogenic and clonogenicity differentiation potential (Lee et al., 2014). Overexpression of miR‐335 leads to cellular senescence of MSCs as evidenced by increased SA‐β‐gal activity and p16 protein level and decreased cell proliferation, impairing the immunomodulatory capacity, and differentiation capacity (Tome et al., 2014). Importantly, Onodera et al. (2017) reported that the expression level of miR‐155‐5p is significantly enhanced in MSCs isolated from aged mice compared with MSCs collected from young mice. Consistent with these findings, we observed that miR‐155‐5p was significantly increased in human AMSCs and serum from aging donors in the current study, suggesting that miR‐155‐5p may be a potential factor in regulating MSC senescence. We further found that overexpressing miR‐155‐5p in YMSCs enhanced the cellular senescent phenotype, including increased SA‐β‐gal activity and expression of p21 and p53 and decreased Ki67‐positive cells. Furthermore, the angiogenic capacity of CdM from miR‐155‐5p‐treated YMSCs was also downregulated. In contrast, inhibition of miR‐155‐5p in AMSCs reduced SA‐β‐gal activity and increased cell proliferation and angiogenesis. Transplantation of anti‐miR‐155‐5p‐AMSCs had a better capacity to attenuate cardiac remodeling and restore heart function in mice following infarction than transplantation of AMSCs. These findings confirmed that miR‐155‐5p accelerated MSC senescence and that inhibition of miR‐155‐5p rejuvenated AMSCs. The exact mechanism underlying miR‐155‐5p regulation of MSC senescence, however, is still largely unknown.

Mitochondrial fusion and fission are essential to maintain cell function, and abnormal mitochondrial dynamics accelerate cellular senescence (Rizza et al., 2018). Our previous study showed that late passage MSCs exhibited large tubular mitochondria compared with early passage MSCs, suggesting that mitochondrial fusion contributes to replicative cellular senescence. Furthermore, knockdown of FGF21 in the early passage MSCs accelerated mitochondrial fusion, leading to cellular senescence (Li et al., 2019). In the current study, we found that AMSCs also exhibited large tubular mitochondria accompanied by decreased p‐Drp1 (Ser616) and increased Mfn2 levels, suggesting that an imbalance of mitochondrial dynamics mediates the physiological senescence of MSCs. Furthermore, treatment with a miR‐155‐5p mimic‐induced mitochondrial fusion and senescence of YMSCs, and these effects were abrogated in part by Mfn2‐siRNA, suggesting that miR‐155‐5p induced MSC senescence by regulating mitochondrial dynamics. Accumulating evidence has shown that AMPK signaling regulates mitochondrial dynamics (Hang et al., 2018; He et al., 2019). Whether miR‐155‐5p mediates mitochondrial fusion by regulating AMPK signaling remains unclear. We sought to identify miR‐155‐5p targets and found that Cab39 is a direct target gene of miR‐155‐5p. It has been reported that Cab39, an upstream coactivator of AMPK, exhibits cell protective functions (Kuwabara et al., 2015). In the current study, we found that the expression of Cab39 and p‐AMPK was greatly reduced in AMSCs compared with YMSCs, suggesting that Cab39/AMPK signaling is associated with the cellular senescence of MSCs. Furthermore, miR‐155‐5p mimic treatment greatly downregulated the expression of Cab39 and p‐AMPK. Combined with miR‐155‐5p mimic‐induced mitochondrial fusion, we sought that miR‐155‐5p‐induced mitochondrial fusion may be regulated by Cab39/AMPK signaling. Moreover, we found that AICAR, an AMPK activator, partially rescued miR‐155‐5p‐induced mitochondrial fusion. These results further confirmed that miR‐155‐5p induces mitochondrial fusion by partially targeting Cab39/AMPK signaling. However, AMPK possess various bypass signaling cascades to regulate its function. In addition to Cab39, we cannot exclude that miR‐155‐5p regulates AMPK activation via other pathways or molecules.

There are some limitations in the current study. First, only miR‐155‐5p in AMSCs was investigated. The functions of other miRNAs that are significantly enriched in AMSCs require further investigation. Second, it has not been investigated yet whether miR‐155‐5p regulates other targets to mediate MSC senescence in addition to Cab39. Third, the long‐term impact of anti‐miR‐155‐5p‐AMSC on heart function recovery following infarction was not examined in this study. Fourth, it would be important to confirm the alteration of miR‐155‐5p‐dependent biological pathways also in heart tissue of MI. Last but not least, more strong and more nonbiased data such as phenotype observation of Cab39‐KO/TG mice and omics‐based screening are needed to verify our findings in the future study.

In summary, our study demonstrates that inhibition of miR‐155‐5p, partially via the Cab39/AMPK signaling pathway, rejuvenates aged MSCs by regulating mitochondrial dynamics and provides a candidate target to enhance the cardioprotection of MSCs for the aged heart following infarction.

## 4 EXPERIMENTAL PROCEDURES

### 4.1 Cell culture

Human YMSCs and AMSCs were isolated from the bone marrow of young and aged volunteer donors, respectively, as previously described (Liang et al., 2019). Written informed consent was obtained from all donors. This study was approved by the research ethics board of Shanghai East Hospital (No. 2016‐050). YMSCs and AMSCs were cultured in DMEM/high glucose (Gibco, 11965084) supplemented with 10% FBS (Life Technologies, 16000), 5 ng/ml EGF (PeProTech, AF‐100‐15), and 5 ng/ml FGF2 (PeProTech, 100‐18B) at 37°C in a humidified atmosphere with 5% CO_2_. The same cell numbers of both YMSCs and AMSCs were plated and passaged at 3‐day intervals. YMSCs and AMSCs at passages 3–4 were used in the current study. HUVECs were grown in RPMI 1640 (Gibco, C11875500BT) supplemented with 10% FBS. 293T cells were grown in DMEM/high glucose (Gibco, 11965084) supplemented with 10% FBS.

### 4.2 Characterization of MSCs

The surface markers of both YMSCs and AMSCs were evaluated by flow cytometry after staining with the following antibodies: anti‐CD73 (BioLegend, 344003), anti‐CD90 (BioLegend, 328107), anti‐CD105 (BioLegend, 323205), anti‐CD31 (BioLegend, 303111), and anti‐CD45 (BioLegend, 304011). The capacity of MSCs to differentiate into adipocytes and osteocytes was evaluated as previously reported (Lian, Zhang, Liang, Gao, & Tse, 2016).

### 4.3 SA‐β‐gal assay

The cellular senescence of MSCs was assessed by a SA‐β‐gal staining kit (Beyotime, C0602). Briefly, MSCs with different treatments were cultured in 6‐well plates. After washing with PBS three times, MSCs were fixed for 20 min and then incubated with the SA‐β‐gal staining solution at 37°C overnight. Finally, SA‐β‐gal‐positive cells, stained blue, were randomly imaged. The percentage of senescent MSCs was evaluated by the ratio of positive MSCs to the total number of MSCs obtained from five different fields of view. The experiments were repeated at least three times.

### 4.4 Scratch wound assay

YMSCs and AMSCs were cultured in a 6‐well plate with complete culture media until they were 100% confluent. Then, scratches of the same width cross the entire well were made using a 200‐µl pipette tip. Subsequently, MSCs were carefully washed with PBS to remove cell debris and then incubated with serum‐free medium in an incubator with 5% CO_2_ at 37°C. After 24 hr of incubation, the migration of MSCs into the wound area was examined. The experiments were repeated at least three times.

### 4.5 Transfection of miR‐155‐5p inhibitor and mimic

miR‐155‐5p mimics, inhibitors, and a negative control (miR‐control) were purchased from GenePharma. In brief, 1 × 10^6^ of MSCs was plated in 10cm plate and then transiently transfected with 50 nM miR‐155‐5p mimics, inhibitors or miR‐control using Lipofectamine 2000 transfection reagent (Invitrogen, 11668027) according to the protocol, respectively. The MSCs were incubated at a 37°C and 5% CO_2_ incubator for 48 hr for transfection and then harvested for further experiments. The transfection experiments were repeated at least three times.

### 4.6 PCR

Total RNA from MSCs or serum was isolated with TRIzol reagent followed by treatment with RNase‐free DNase I (Takara, 2270A). Reverse transcription was performed using a PrimeScript RT Reagent Kit (Takara, RR037A). For miRNA expression detection, Taqman miRNA assays were used to quantify the expression levels of mature miR‐155‐5p (002623, Applied Biosystems). The relative expression level of microRNAs was normalized by U6 (001973, Applied Biosystems). The reactions were performed in 7500 Fast Real‐Time System (Applied Biosystems), and the reaction mix was incubated at 95°C for 30 s, followed by 40 cycles of 95°C for 8 s and 60°C for 30 s. The expression of miR‐155‐5p was normalized to the expression of U6 using the 2−ΔΔCt cycle threshold method. The experiments were repeated at least three times. Moreover, to examine MSC survival after transplantation in the heart tissue, DNA was extracted from paraffin‐embedded tissues according to the manufacturer's protocols (Tiangen, DP316). Human Alu‐sx repeat sequences were detected by genomic PCR. The primer of Alu‐sx was: F:5′‐GGCGCGGTGGCTCACG‐3′, R:5′‐TTTTTTGAGACGGAGTCTCGCTC‐3. Finally, the product was evaluated by electrophoresis in 2.0% agarose gel supplemented with ethidium bromide.

### 4.7 Preparation of CdM derived from MSCs

Both YMSCs and AMSCs were seeded into 6‐well plates with growth medium and cultured until 70%–80% confluence. After different treatments, the medium was replaced with 2 ml per well serum‐free medium. After 48 hr, the CdM was harvested, centrifuged, filtered, and stored at −80°C until use.

### 4.8 HUVEC tube formation assay

The angiogenic capacity of CdM from MSCs was assessed by the capillary tube formation assay. Briefly, HUVECs (30,000 cells/well) were seeded in a 96‐well plate coated with growth factor‐reduced Matrigel (BD Biosciences, 356230). Next, HUVECs were treated with CdM from YMSCs, AMSCs, YMSCs transfected with miR‐155‐5p mimic or AMSCs transfected with miR‐155‐5p inhibitor. After 6 hr of treatment, capillary‐like tube formation was imaged. Tube length and number of branches were analyzed using ImageJ software. The experiments were repeated at least three times.

### 4.9 MitoTracker staining

The morphology of mitochondria in MSCs was detected by MitoTracker Green FM (Invitrogen, M7514) according to the manufacturer's protocol. Briefly, MSCs were cultured in 24‐well plates with cover slides and then treated according to experimental settings. Subsequently, MSCs were washed and incubated with DMEM supplemented with 20 nM MitoTracker Green FM for 15 min at room temperature. Finally, after washing with PBS three times, MSCs were imaged using a confocal microscope.

### 4.10 Western blotting

Total protein of treated MSCs was extracted using a total protein extraction kit (Bestbio, BB‐3101), and the concentrations were measured by a bicinchoninic acid (BCA) assay kit (Thermo, 231227). A total of 30 μg protein was resolved by 10% Tris‐glycine gel electrophoresis and then transferred onto a PVDF membrane. After blocking with 5% fat‐free milk in TBST, the PVDF membranes were incubated overnight at 4°C with the following antibodies: anti‐p53 (Abcam, ab26), anti‐p21 (Abcam, ab109199), anti‐p‐Drp1 (Ser616) (CST, 3455), anti‐Drp1 (CST, 14647), anti‐Mfn2 (Abcam, ab124773), anti‐Mfn1 (Abcam, ab57602), anti‐p‐AMPK (CST, 4184), anti‐AMPK (CST, 5832), anti‐Cab39 (Abcam, ab51132), and anti‐GAPDH (CST, 2118). Next, the membranes were washed three times with TBST and incubated with secondary antibodies (1:1,000, CST) at room temperature for 1 hr and then exposed in a dark room. The quantification of Western blotting was analyzed using ImageJ software (National Institutes of Health, Bethesda, MD, USA) in three independent experiments.

### 4.11 Immunofluorescence staining

Mesenchymal stem cells were fixed with 4% paraformaldehyde (P0099, Beyotime) for half an hour and permeabilized with 0.1% Trion X‐100 in PBS. After washing with PBS twice, the cells were blocked with 10% BSA and then incubated with anti‐ki‐67 antibody (Abcam, ab15580) overnight at 4°C. Subsequently, the cells were washed and incubated with the appropriate secondary antibodies conjugated with fluorophores (1:1,000). Finally, cover slides were mounted with DAPI and imaged. The percentage of ki‐67‐positive cells was calculated as the ratio of ki‐67‐positive MSCs to all DAPI‐positive cells ×100%. The experiments were repeated at least three times.

### 4.12 Luciferase assay

The 3′‐UTR of human Cab39 was inserted into the pGL3 luciferase reporter vector (Promega, Madison, WI, USA). Mutations in the seed region of the miR‐155‐5p‐binding site in the Cab39 3′‐UTR were generated by overlap extension PCR. 293T cells were cultured in 24‐well plates and then cotransfected with wild‐type pGL3‐Cab39‐3′‐UTR or mutant Cab39‐3′‐UTR and a scrambled miRNA control or miR‐155‐5p mimics by Lipofectamine 2000 (Invitrogen, 11668027). Forty‐eight hours after transfection, the luciferase activities were determined by the Dual‐Luciferase Reporter Assay System Kit (E1910, Promega) according to the manufacturer's protocol. The experiments were repeated at least three times.

### 4.13 Viral vector construction and infection

The lentiviral plasmid constructs for the inhibition of miR‐155‐5p in AMSCs were purchased from GenePharma. The plasmid contained an expression cassette consisting of a CMV promoter followed by cDNA encoding eGFP and an anti‐miR155‐5p sequence (Figure S7). The lentivirus was packaged as previously reported (Liang et al., 2019). For stable transduction, AMSCs at a confluence of 70%–80% were infected by lentivirus at a multiplicity of infection of 10 with polybrene (8 μg/ml). The infection efficiency was determined by eGFP fluorescent signal viewed under the microscope at 48 hr after infection and the cells were labeled as anti‐miR‐155‐5p‐AMSCs.

### 4.14 TEM

The morphology of mitochondria was examined by TEM. Briefly, MSCs were fixed with 2.5% glutaraldehyde in phosphate buffer for 4 hr and then postfixed with 1% OsO4 for 2 hr. Next, after dehydration with a graded concentration of ethanol, MSCs were infiltrated with 1:1 acetone: Spurr resin (SPI‐CHEM, 02690‐AB) for 1 hr at room temperature, 1:3 acetone: Spurr resin for 3 hr, and then absolute Spurr resin overnight. Finally, electronic images were captured using a TEM (Hitachi, H‐7650).

### 4.15 MI model and transplantation of MSCs

All animal procedures in the current study were approved by the Committee on the Use of Live Animals in Teaching and Research (CULTAR) of the Tongji University for Laboratory Animal Medicine (No. TJLAC‐019‐134). Female C57/B6J mice, 12 months of age, were used to induce an acute MI model by ligation of the left anterior decedent coronary artery (LAD) as previously described (Liang et al., 2019). After LAD ligation, mice randomly received one of the following treatments: (a) phosphate‐buffered saline (PBS) (MI group, *n* = 12); (b) 3 × 10^5^ YMSCs (YMSC group, *n* = 11); (c) 3 × 10^5^ AMSCs (AMSC group, *n* = 12) or 3 × 10^5^ anti‐miR‐155‐5p‐aged MSCs (anti‐miR‐155‐5p‐AMSC group, *n* = 12). All MSCs were suspended in 100 μl PBS and were injected intramuscularly at four sites around the border zone of the infarcted heart. Another group of mice that underwent thoracotomy without LAD ligation served as the control group (Sham group, *n* = 6). Cardiac function in each mouse was assessed by transthoracic echocardiography (Ultramark 9; Soma Technology) at baseline (before MI), 1, or 28 days following MI. LVEF and LVFS were calculated as previously described (Liao et al., 2019).

### 4.16 Masson's staining

After echocardiography assessment at 28 days post‐MI, all the mice were sacrificed, and the heart tissues were harvested, embedded, and sectioned. The infarction size of the mouse heart, as evidenced by fibrosis, was examined by Masson's staining kit according to the manufacturer's protocol (HT15, Sigma). The percent infarct size was calculated as the ratio of fibrosis area to total LV area ×100%.

### 4.17 TUNEL staining

The apoptosis of cardiomyocytes in the heart tissue from different groups was evaluated by TUNEL staining as previously described (Zhang et al., 2015). The sections were mounted with 4′, 6‐diamidino‐2‐phenylindole (DAPI; Vector Laboratories, Inc.) and imaged using a fluorescence microscope. The apoptotic rate was calculated as the ratio of TUNEL‐positive cells to DAPI‐positive cells ×100%.

### 4.18 Immunohistochemistry

To determine the blood vessel density in the heart tissue from different groups, the heart sections were immunohistochemically stained with anti‐CD31 (1:100; ab19898, Abcam) and anti‐α‐smooth muscle actin (α‐SMA) (1:100; ab5694, Abcam). The capillary densities and arteriole densities were expressed as the average number of CD31‐ or α‐SMA‐positive blood vessels per field (×10).

### 4.19 Statistical analysis

All data are expressed as the mean ± *SEM*. Statistical analyses were performed using Prism 5.04 software (GraphPad Software for Windows). Comparisons between two groups were analyzed by unpaired Student's *t* test, and comparisons between more than two groups were analyzed by one‐way ANOVA followed by Bonferroni test. A value of *p* < .05 was considered statistically significant.

## CONFLICT OF INTEREST

The authors confirm that they have no conflicts of interest.

## AUTHOR CONTRIBUTIONS

Y. Zhang, X. Li and X. Liang designed the research, analyzed the data and wrote the manuscript. Y. Hong performed the research, analyzed the data and wrote the manuscript. H. He, G. Jiang, H. Zhang and W. Tao contributed to performing the research and preparing experimental reagents. Y. Ding, J. Liu, D. Yuan, H. Fan and F. Lin helped to analyses the data and provided the materials.

## Supporting information

Figures S1–S7
