# Supplementary material for: miR‐155‐5p inhibition rejuvenates aged mesenchymal stem cells and enhances cardioprotection following infarction
Source: Aging Cell. 2020 Mar 20;19(4):e13128. doi: 10.1111/acel.13128 (PMC7189985; doi:10.1111/acel.13128)
Supplement: Supplementary file 1 — Figures S1–S7 [file ACEL-19-e13128-s001.docx]

**Supplemental information**

**Supplemental Figure 1. Characterization of YMSCs and AMSC.**

(A) Surface marker profiling of YMSCs and AMSCs was evaluated by flow cytometry. Both YMSCs and AMSCs expressed CD73, CD90 and CD105; but not CD31 and CD45. (B) Adipogenic differentiation determined by Oil red staining and quantification of adipogenic efficiency in YMSCs and AMSCs. Scale bar=200 μm. (C) Osteogenic differentiation determined by Alizarin red staining and quantification of osteogenic efficiency in YMSCs and AMSCs. Scale bar=200 μm. (D) Representative images of wound healing assay showing the migratory capacity and quantification of wound recovery rate of YMSCs and AMSCs. Scale bar=200 μm. Data are expressed as mean±SEM. n=3. **p<0.05, ***p<0.001.*

*
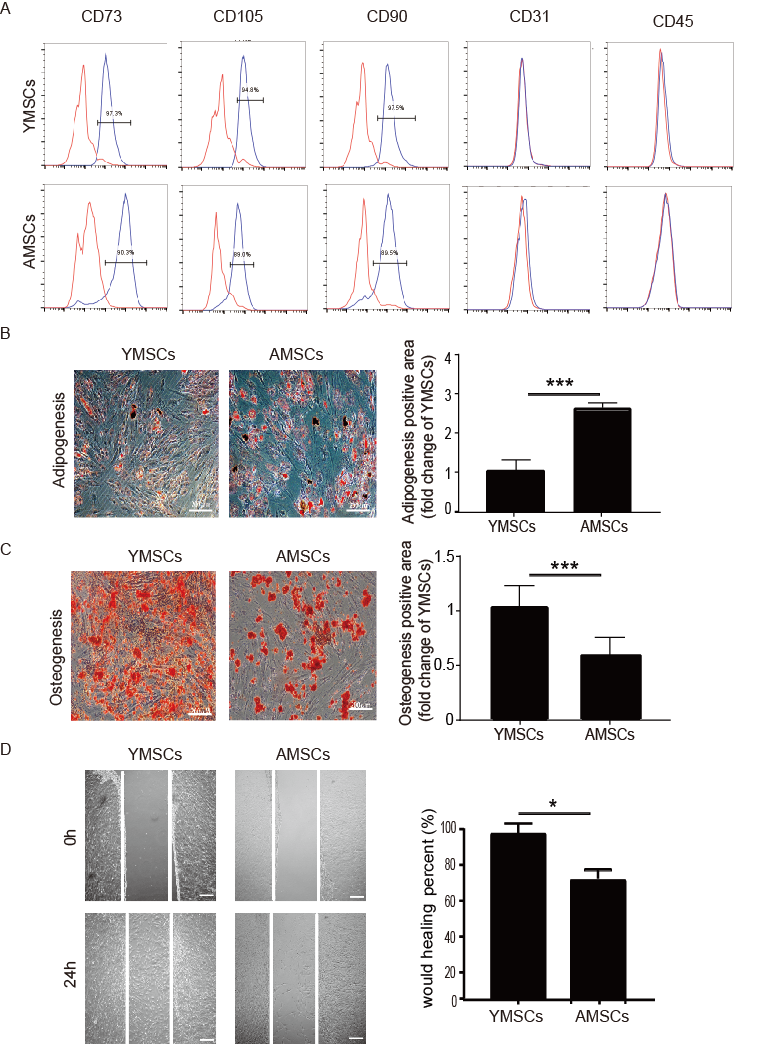
*

**Supplemental Figure 2. miR-155-5p regulated cell proliferation and paracrine effects of YMSCs and AMSCs**

(A) The level of miR-155-5p expression was upregulated in miR-155-5p mimic-treated YMSCs compared with miR-control-treated YMSCs. (B) Immunostaining of the proliferation marker Ki67 and quantitative analysis of Ki67 positive cells in miR-control or miR-155-5p mimic-treated YMSCs. Scale bar=100 μm. (C) Representative images of tube formation and analysis of tube length of HUVECs received CdM treatment from miR-control or miR-155-5p mimic-treated YMSCs. Scale bar=200 μm. (D) The level of miR-155-5p expression was downregulated in miR-155-5p inhibitor-treated AMSCs compared with miR control-treated AMSCs. (E) Immunostaining of the proliferation marker Ki67 and quantitative analysis of Ki67 positive cells in miR-control or miR-155-5p inhibitor-treated AMSCs. Scale bar=100 μm. (F) Representative images of tube formation and analysis of tube length of HUVECs received CdM treatment from miR-control or miR-155-5p inhibitor-treated AMSCs. Scale bar=200 μm. Data are expressed as mean±SEM. n=3. **p<0.05;**p<0.01; ***p<0.001.*


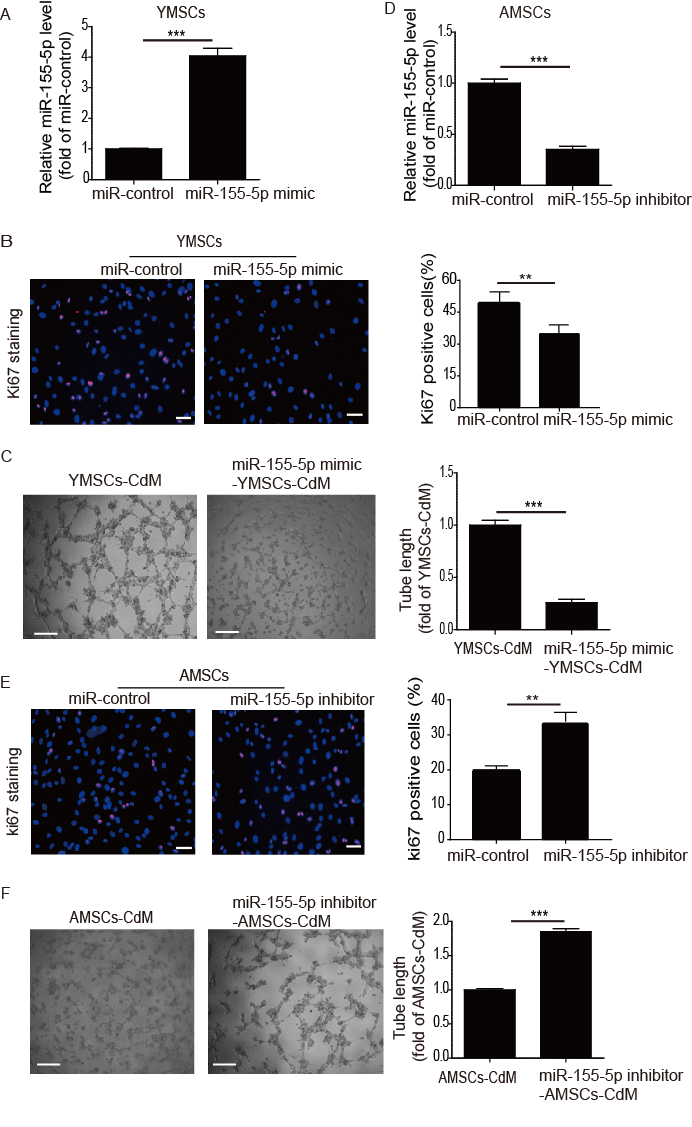


**Supplemental Figure 3. Inhibition of miR-155-5p ameliorated the cellular senescence of AMSCs via activating mitochondrial fission and inhibiting mitochondrial fusion**

*
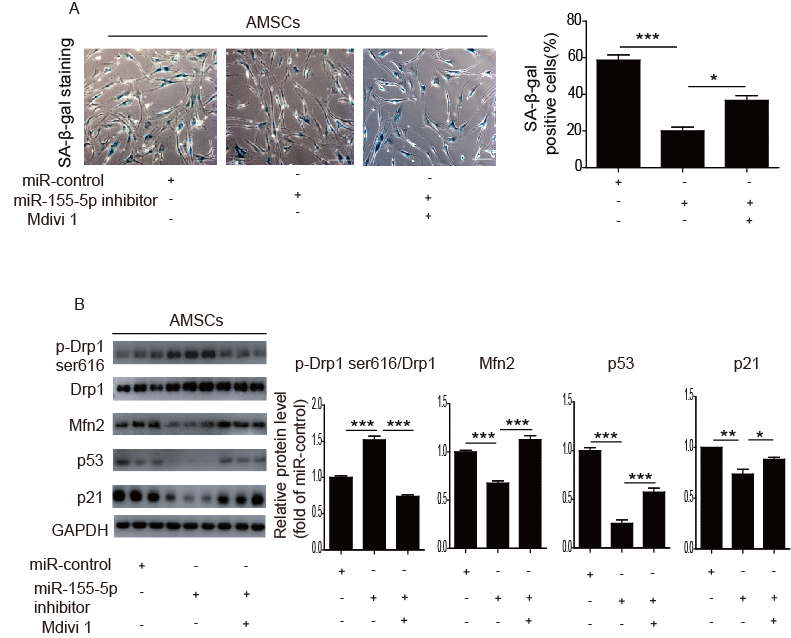
*(A) Representative images of SA-β-gal staining and quantitative analysis of SA-β-gal positive cells in miR-control, miR-155-5p inhibitor or miR-155-5p inhibitor+Mdivi 1-treated AMSCs. Scale bar=200 μm. (B) Western blotting and quantitative analysis of the expression level of p-Drp1 ser616, Mfn2, p21 and p53 in miR-control, miR-155-5p inhibitor or miR-155-5p inhibitor+Mdivi 1-treated AMSCs. Data are expressed as mean±SEM. n=3. **p<0.05;**p<0.01; ***p<0.001.*

**Supplemental Figure 4. The luciferase activity of YMSCs and AMSCs was examined.** YMSCs and AMSCs were transfected with wild-type pGL3-Cab39-3’-UTR luciferase reporter and then the luciferase activity was examined. Compared with YMSCs, the luciferase activity was dramatically reduced in AMSCs. Data are expressed as mean±SEM. n=3. ****p<0.001.*


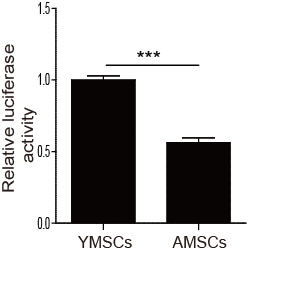


**Supplemental Figure 5. Cab39/AMPK signaling pathway was involved in MSCs senescence.** (A) miR-155-5p mimic treatment significantly reduced the mRNA level of Cab39 in YMSCs. (B) Western blotting and quantitative analysis of the expression level of Cab39 and p-AMPK in YMSCs and AMSCs. Data are expressed as mean±SEM. n=3. ****p<0.001.*

*
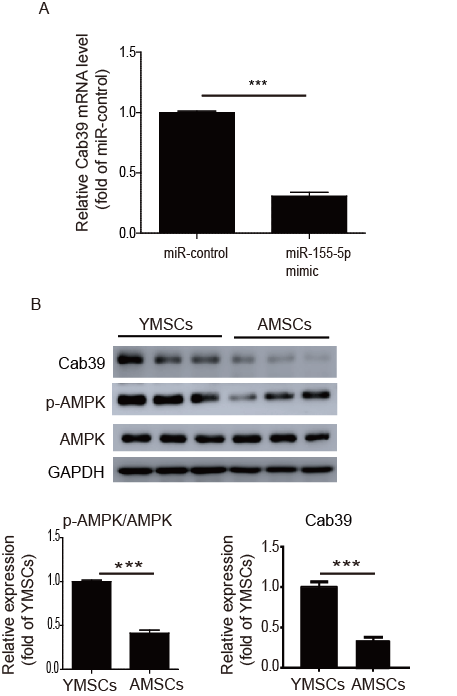
*

*
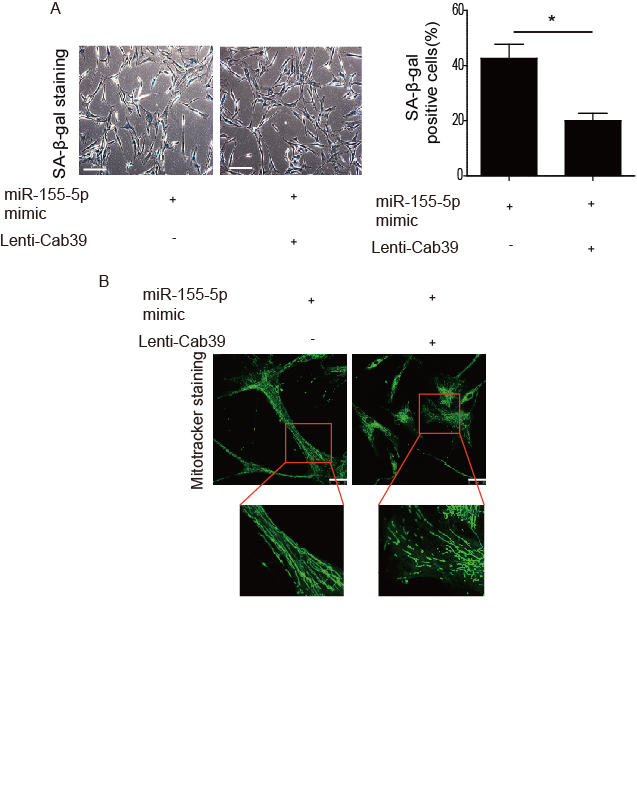
***Supplemental Figure 6. Overexpressed Cab39 rescued miR-155-5p-induced YMSC senescence and inhibited mitochondrial fusion.** (A) Representative images of SA-β-gal staining and quantitative analysis of SA-β-gal-positive cells in miR-155-5p-treated or miR-155-5p+Lenti-Cab39-treated YMSCs. Scale bar=200 μm (B) Representative images of MitoTracker staining of miR-155-5p-treated or miR-155-5p+Lenti-Cab39-treated YMSCs. Scale bar=50 μm. Data are expressed as mean±SEM. n=3. **p<0.05.*

**
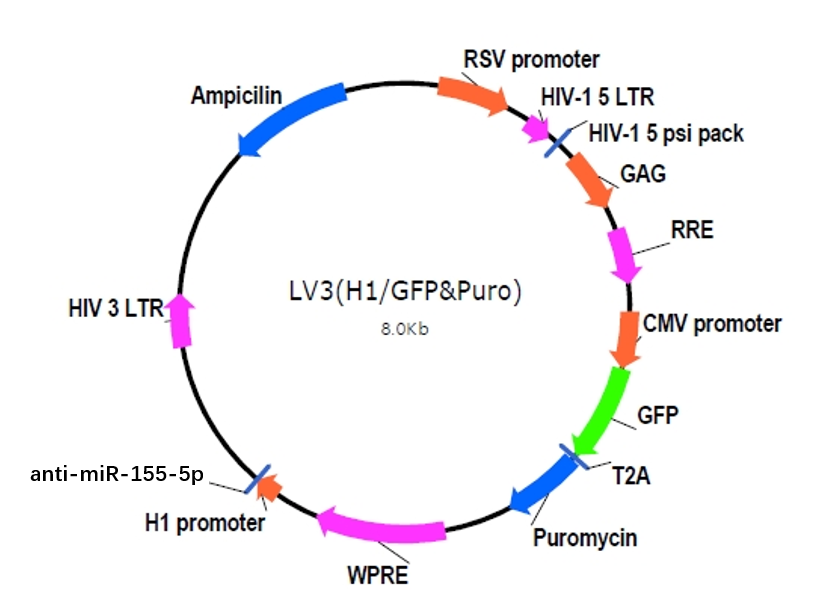
Supplemental Figure 7. Structures of Lenti-miR-155-5p inhibitor which contain GFP reporter gene.**
